# Heartbreaking Decisions: The Dogma and Uncertainties of Antimicrobial Therapy in Infective Endocarditis

**DOI:** 10.3390/pathogens12050703

**Published:** 2023-05-12

**Authors:** Jennifer L. Adema, Aileen Ahiskali, Madiha Fida, Krutika Mediwala Hornback, Ryan W. Stevens, Christina G. Rivera

**Affiliations:** 1 Department of Pharmacy, East Carolina University Health, Greenville, NC 27834, USA; 2 Department of Pharmacy, Hennepin Healthcare, Minneapolis, MN 55415, USA; aileen.ahiskali@hcmed.org; 3 Division of Public Health, Infectious Diseases and Occupational Medicine, Mayo Clinic, Rochester, MN 55902, USA; fida.madiha@mayo.edu; 4 Department of Pharmacy, Medical University of South Carolina (MUSC) Health, Charleston, SC 29425, USA; mediwala@musc.edu; 5 Department of Pharmacy, Mayo Clinic, Rochester, MN 55902, USA; stevens.ryan@mayo.edu

**Keywords:** infective endocarditis, antimicrobial stewardship, *Staphylococcus aureus*, novel antimicrobials

## Abstract

Infective endocarditis (IE) is a rare but increasingly prevalent disease with high morbidity and mortality, requiring antimicrobials and at times surgical intervention. Through the decades of healthcare professionals’ experience with managing IE, certain dogmas and uncertainties have arisen around its pharmacotherapy. The introduction of new antimicrobials and novel combinations are exciting developments but also further complicate IE treatment choices. In this review, we provide and evaluate the relevant evidence focused around contemporary debates in IE treatment pharmacotherapy, including beta-lactam choice in MSSA IE, combination therapies (aminoglycosides, ceftaroline), the use of oral antimicrobials, the role of rifamycins, and long-acting lipoglycopeptides.

## 1. Introduction

Infective endocarditis (IE) is a life-threatening disease that global healthcare professionals have been both treating and attempting to prevent for decades. The incidence has increased and is estimated to currently be 2–12 cases per 100,000 people [1,2]. Some theories of why incidence continues to rise include the increased use of implantable cardiac devices, a greater number of patients undergoing hemodialysis for end-stage renal disease, increasing numbers of persons who inject drugs (PWID), and more patients with congenital heart diseases surviving to adulthood [3]. Despite years of scientific research and clinical experience, IE remains a clinical challenge in many cases. While IE and bacteremias share similar portals of entry and initial disease state progression, IE is complicated by bacterial adhesion, colonization, and vegetation formation on the cardiac valve [4]. In particular, this biofilm formation can decrease the ability for antimicrobials to completely eradicate the infection due to the high bacterial density present, making antimicrobial penetration difficult [5]. Even when valve replacement surgery can be utilized to achieve source control, effective and safe antimicrobials are also required.

Biofilms play a critical role in the complexity and persistence of IE. There is evidence to suggest that both native and prosthetic IE infections are biofilm-related [6]. The treatment of IE is a significant challenge due to the complex and persistent nature of the biofilm-embedded micro-organisms. 

Biofilms are microcolonies of bacteria embedded in an extracellular matrix consisting of exopolysaccharides, extracellular DNA (eDNA), proteins, and other compounds. The bulk of the bacterial burden in IE resides in the biofilm, and planktonic bacteria in the blood make up a very small proportion of the bacterial burden. 

The unique environment of the biofilms protects pathogens from the host immune system as well as antimicrobials [7]. First, the biofilm matrix acts as a physical barrier for the antimicrobials. In addition, the antimicrobial agents may be bound by the matrix (e.g., positively charged aminoglycosides may bind to eDNA). In the deeper layers of biofilms, changes in the availability of nutrients and oxygen may lead to metabolic slowing or metabolic inactivity, which affects the activity of cell wall active agents such as beta-lactams. In fact, subinhibitory levels of beta-lactams may induce biofilm production [8]. In addition, the eDNA in the matrix may promote horizontal gene transfer and thus the uptake of resistance genes by bacteria [9]. While several antimicrobial agents, such as rifampin and quinolones, have been shown to have an impact on infections involving biofilms, the timing of initiation of treatment is critical. In general, starting treatment early, before the biofilm has fully matured, appears to be more effective than starting later [10].

Thus, the best selection for antimicrobial(s) involves understanding the unique pathophysiology of IE, the interplay with drug pharmacokinetics and dynamics, adverse drug reaction risks, and the limitations of available clinical evidence. With these multiple complexities and existing gaps in evidence, certain IE treatment approaches are subject to debate. In this narrative review, we explore challenging IE treatment decisions that frequently arise for practicing clinicians, moving from longer-standing issues (beta-lactam selection and rifampin use for staphylococcal IE, aminoglycoside combination for Gram-positive IE), to contemporary controversies (daptomycin and ceftaroline combination for Gram-positive IE and oral antimicrobials), and then lastly discussing newer drugs challenging the status quo in IE (long-acting lipoglycopeptide antibiotics).

## 2. Beta-Lactam Selection in Methicillin-Susceptible *Staphylococcus aureus* IE

*Staphylococcus aureus* is one of the most common pathogens associated with both native and prosthetic valve IE [11]. The treatment of IE due to *S. aureus* is primarily dependent upon the organism’s methicillin susceptibility. Methicillin-resistant *S. aureus* (MRSA) has historically garnered significant attention, given its pathogenicity and limited treatment options, with the drugs of choice being vancomycin and daptomycin [11,12]. Yet, amongst clinical isolates of *S. aureus*, methicillin-susceptible (MSSA) strains often represent the epidemiologic majority [13,14]. Despite the availability of several antimicrobials with activity against MSSA, the optimal therapy has long been debated.

Though study cohorts on the topic generally include few patients with endocarditis, it is well established that definitive treatment of MSSA BSI with vancomycin, as compared to beta-lactam based therapies, is associated with higher rates of adverse outcomes (e.g., mortality) [15,16,17]. It is unsurprising that the Infectious Diseases Society of America/American Heart Association (IDSA/AHA) IE diagnosis and management guidelines recommend beta-lactams (i.e., antistaphylococcal penicillins (ASP) and cefazolin) as first-line therapy for MSSA IE, even indicating careful allergy assessment and consideration of beta-lactam desensitization in patients with IgE-mediated beta-lactam allergies [11]. However, several beta-lactams have some level of antistaphylococcal activity, and the controversy therein lies: which agents should be preferentially selected over others?

### 2.1. Beta-Lactam/Beta-Lactamase Inhibitor Combinations (BL/BLIs)

The efficacy of BL/BLIs as compared to other beta-lactam therapies for MSSA bacteremia has been evaluated in a few small retrospective cohorts [18,19]. These data largely suggest that the use of BL/BLIs, such as piperacillin/tazobactam, is associated with higher rates of mortality than treatment with cefazolin or ASPs, though, as seen with most data comparing beta-lactams for this syndrome, the number of patients with IE was limited (<10%). Specifically, one propensity-score matched study of patients with MSSA bacteremia demonstrated that patients treated with ASPs or cefazolin (*n* = 48) had a 30-day mortality hazard ratio of 0.1 (95% CI 0.01–0.78) as compared to BL/BLI treated patients (*n* = 48) [18]. Similarly, Paul et al. performed a study on patients with MSSA bacteremia, which demonstrated a 2.68 (95% CI 1.23–5.85) adjusted odds ratio for 30-day mortality in patients treated with piperacillin/tazobactam as compared to cloxacillin or cefazolin [19]. 

### 2.2. Ceftriaxone/Cefotaxime

The “optimal selection” controversy becomes more palpable when the focus is shifted to third-generation cephalosporins. Infectious disease dogma has historically created uncertainty around the use of third-generation cephalosporins to manage serious MSSA infections. Ceftriaxone or cefotaxime was compared to cloxacillin or cefazolin in a retrospective cohort study [19]. Similar to BL/BLIs, ceftriaxone was associated with increased odds of 30-day mortality when compared to cloxacillin/cefazolin (aOR 2.24, 95% CI 1.23–4.08). This aligns with an abstract that demonstrated ceftriaxone-treated patients (*n* = 37) with MSSA bacteremia had higher rates of 30-day (8.1% vs. 3.8%) and 90-day (27% vs. 8.6%) treatment failure when compared to a cohort (*n* = 186) treated with cefazolin and ASPs [20]. Similarly, a small, retrospective study within the veterans affairs system of 71 patients treated with either ceftriaxone or cefazolin for MSSA bacteremia found ceftriaxone to be associated with a higher rate of treatment failure (54.5% vs. 28.9%, *p* = 0.029), though they only included seven patients with IE [21].

Conversely, a study evaluating outcomes of cefazolin (*n* = 161) versus ceftriaxone (*n* = 87) found no difference in the primary outcome of clinical cure (86.2% ceftriaxone vs. 90.1% cefazolin, *p* = 0.359). This finding held true after adjustment for Charlson comorbidity index and Pitt bacteremia score; however, only 7.7% of patients included had IE [22]. One retrospective study evaluated the composite outcome of 90-day all-cause mortality, readmission due to MSSA infection, and microbiologic failure for patients with MSSA bacteremia being discharged on ceftriaxone versus oxacillin or cefazolin [23]. In the full cohort, they found no difference with regard to the primary outcome (19% ceftriaxone vs. 21% cefazolin/oxacillin, *p* = 0.7). Interestingly, this study included 83 patients with suspected IE (28.4% in ceftriaxone group vs. 43.2% in cefazolin group). A subgroup analysis of IE treated with ceftriaxone (*n* = 42) compared to cefazolin or oxacillin (*n* = 41) demonstrated ceftriaxone to be associated with a numerically higher rate of both the primary composite outcome (25.6% vs. 10%, *p* = 0.17) and 90-day all-cause mortality (14.3% vs. 2.4%, *p* = 0.11) [23]. Most recently, a systematic review and meta-analysis evaluated the use of ceftriaxone (*n* = 1037) compared to standard of care (SOC) (*n* = 2088) therapies (i.e., ASPs and cefazolin) in MSSA bacteremia [24]. It did not identify a statistically significant difference between groups for clinical cure, microbiologic cure, 30-day/90-day mortality, 90-day hospital readmission, or adverse drug reaction (ADR) occurrence. Only 7 of the 12 studies included enrolled patients with IE (11.5% of total cohort with IE), and the treatment distribution was routinely skewed toward the SOC arm. As a result, the authors concluded that the findings could not be extrapolated to IE given the underrepresentation in the population [24].

Lastly, it remains worthy of consideration that ceftriaxone pharmacokinetics (PK) and pharmacodynamics (PD) may play a role in the above heterogeneous findings. The predominant dosing used in the above-referenced studies was 1–2 g intravenous (IV) every 24 h, with 2 g IV every 24 h representing the most common dose. A higher dose of 2 g IV every12 h was rarely employed. A recent hollow fiber PD study found that, for an isolate with a ceftriaxone minimum inhibitory concentration (MIC) of 4 mcg/mL, only a dosing regimen of 2 g IV every 12 h achieved sustained bacterial death. The authors propose that at routine doses, ceftriaxone PK/PD may be insufficient [25].

### 2.3. Cefazolin vs. ASPs

Perhaps the most significant controversy related to optimal beta-lactam therapy selection for MSSA IE involves selecting between cefazolin and ASPs. IDSA/AHA guidelines recommend ASPs as first-line therapy for both native valve endocarditis (NVE) and prosthetic valve endocarditis (PVE), listing cefazolin as an alternate option for those patients with non-immediate-type hypersensitivity reactions to penicillins [11]. This treatment distinction was based on the potential that the cefazolin inoculum effect (CzIE) may render cefazolin less effective than ASPs based on in vitro findings, demonstrating cefazolin is more susceptible to beta-lactamase degradation than ASPs [26]. Specifically, isolates are determined to display CzIE if there is an increase in MIC to >16 mcg/mL when susceptibility testing is performed with higher bacterial inoculums of ~5 × 10^7^ colony-forming units (CFU)/mL, as opposed to the standard density of ~5 × 10^5^ CFU/mL [27]. This testing is not routinely performed in clinical laboratories, given the laborious nature of the methodologies. An epidemiologic study of *S. aureus* isolates from North America identified the CzIE to be displayed in 18.6% (57/305), with some geographical variability in prevalence [27]. However, data pertaining to the impact of CzIE on patient outcomes are limited. One study evaluated the clinical outcomes of cefazolin-treated patients with MSSA bacteremia stratified by the CzIE status of *S. aureus* isolates, with CzIE present in 57.5% (65/113) of patients [28]. Persistent bacteremia was associated with CzIE (9% vs. 0%, *p* = 0.04). Treatment failure at 12 weeks was numerically higher in the CzIE group (48% vs. 25%); however, this finding did not reach statistical significance (*p* = 0.13). Another study performed in Argentina prospectively enrolled 77 cefazolin-treated patients with MSSA bacteremia and screened the isolates for the presence of CzIE [29]. There were 42 patients (54.5%) with isolates demonstrating the effect, and 30-day mortality was higher in patients with CzIE-producing organisms than those without (39.5% vs. 15.2%, *p* = 0.034). Rates of IE were low, at only 10.4% of the total cohort, and were numerically higher in those demonstrating CzIE (14.3% vs. 5.7%). On multivariable analysis, CzIE and secondary sources of bacteremia were found to be associated with a relative risk of 30-day mortality of 2.65 (95% CI 1.10–6.42) and 2.15 (95% CI 1.01–4.57), respectively. The authors concluded that cefazolin may represent suboptimal therapy in patients with high bacterial burdens, uncontrolled sources, and isolates demonstrating CzIE [29].

Other clinical data comparing cefazolin and ASPs in the management of MSSA bacteremia and IE are largely retrospective, and the majority of studies do not test for the presence of CzIE. A retrospective report of 3167 patients from 199 VA hospitals demonstrated cefazolin (*n* = 1163) was associated with a lower rate of 30-day (HR 0.77, 95% CI 0.51–0.78) and 90-day mortality (HR 0.77, 95% CI 0.66–0.90) after adjustment for source, secondary complications (i.e., endocarditis or osteomyelitis), comorbidities, and severity of illness [30]. The study only included 197 (6.2%) patients with IE, with rates of IE of 7% in the ASP group compared to 4% in the cefazolin group (*p* = 0.002). Several systematic reviews/meta-analyses have generated similar findings when comparing cefazolin to ASPs for MSSA bacteremia. These also often demonstrate low rates of patients with IE [31,32,33]. A small study from France compared treatment outcomes specifically in patients with MSSA IE who received either an ASP (*n* = 157) or cefazolin (*n* = 53). The 90-day mortality rate was 24.5% in the cefazolin group as compared to 28.7% in the ASP group (*p* = 0.561), and therapy selection was not associated with 90-day mortality on multivariable analysis [34].

Two other features are often considered when selecting optimal therapy amongst options considered SOC: adverse effects and penetration of the central nervous system (CNS). With regard to adverse effects, the literature has predominantly demonstrated that cefazolin has lower rates of adverse effects (e.g., allergic reactions, hematologic toxicity, electrolyte derangements (e.g., hypokalemia), hepatotoxicity, and nephrotoxicity) and/or related discontinuations [31,32,33,34]. Also of note, within the ASPs, oxacillin has been shown to have a more favorable safety profile than nafcillin, specifically pertaining to lower rates of hypokalemia and nephrotoxicity [35,36]. Given the risk for CNS emboli in left-sided IE, antimicrobial CNS penetration is commonly a factor when selecting definitive beta-lactam therapy. Cefazolin has historically been treated with skepticism when the CNS is involved, given questionable penetration across the blood–brain barrier [37]. Yet, evolving data, including case reports, PK studies, and expert opinions, continue to challenge this dogma. These reports suggest that cefazolin, if used at more aggressive dosing (e.g., 2 g IV every 6 h or 8–10 g/day as continuous infusion) could be considered as an alternative to ASP in infections involving the CNS [37,38,39].

### 2.4. Beta-Lactam Selection for IE Conclusions

At this time, BL/BLIs should not be routinely recommended for definitive management of MSSA bacteremia and, consequently, IE. Similarly, the conflicting evidence and lack of enrollment of IE patients in the available literature makes it difficult to take a strong stance in favor of the use of ceftriaxone in IE; however, if employed, we recommend a ceftriaxone dose of 2 g IV every 12 h in most circumstances. Optimal beta-lactam selection in the management of MSSA IE should be centered around the use of cefazolin or an ASP. Furthermore, with all the evidence considered (summarized in Table 1), cefazolin appears to be at least similarly effective to ASPs in the treatment of MSSA bacteremia and IE. Yet, it is still unclear as to how CzIE may impact outcomes, particularly in isolates demonstrating the presence of the effect and/or deep-seated, high inoculum infections with poor source control. More clinical outcome data are warranted that are specific to IE with strains displaying CzIE as compared to those that do not. This would require large prospective trials including CzIE positive isolates and/or clinical microbiology laboratories to more routinely screen for CzIE to facilitate retrospective outcome comparisons.

## 3. Rifamycins for Staphylococcal IE

### 3.1. Rifampin Pharmacology Relevant to IE

The rifamycins, namely rifampin, garner a highly specific role in IE treatment due to their unique pharmacologic properties. Rifampin kills proliferating extracellular organisms and is highly active against both coagulase-positive and -negative staphylococci. Rifampin binds to the beta subunits of DNA-dependent RNA polymerase, encoded by the rpoB gene, and interferes with protein synthesis by preventing chain initiation [40]. Rifampin is usually bactericidal and demonstrates concentration-dependent killing. Additionally, rifampin has a long post-antibiotic effect, an unusual ability to enter cells, and intracellular antibacterial activity [41,42]. Rifampin has outperformed other antibiotics against staphylococcal biofilms in vitro when tested in combination with another antistaphylococcal agent [43]. Thus, rifampin has advantageous properties for staphylococcal IE: (1) bactericidality against staphylococci, (2) high intracellular levels, (3) biofilm penetration, and (4) wide tissue distribution, including CNS, bone, and joint.

Various antimicrobial combinations with rifampin have been analyzed in experimental models; however, significant heterogeneity precludes direct comparison of various rifampin-based combinations due to differences in employed bacterial inoculum concentrations, antimicrobial concentrations, susceptibility and synergy testing methodology, as well as timing of initiation of and duration of therapy [44]. In general, higher bacterial inoculums have been associated with the emergence of rifampin resistance and failure of therapy [45,46]. In clinical studies, a similar effect has been demonstrated when using active bacteremia as a surrogate for high bacterial burden, as shown in a retrospective study of S. aureus bacteremia by Reidel et al. in which rifampin resistance emerged in 56% of cases when rifampin was initiated during active bacteremia [47]. Hence, expert opinion advocates the use after clearance of bacteremia [48].

On the other hand, a significant disadvantage to rifampin is the requirement to be combined with another active drug. Staphylococci resistance can emerge rapidly with monotherapy; genetic resistance has been identified after a single dose of rifampin with an MRSA isolate [49]. There are four major mechanisms of rifampin resistance by staphylococci: (1) mutations in the rpoB gene altering the binding site for rifampin, thereby reducing its binding affinity; (2) overexpression of efflux pumps, which actively pump out rifampin from the bacterial cell, thus reducing its intracellular concentration; (3) reduced cell and/or membrane permeability, which limits the entry of rifampin into the cell; and (4) acquisition of resistance genes through horizontal gene transfer from other bacteria [50]. Patient fatality has been reported from bacteremia and IE due to MRSA with rapid emergence of rifampicin resistance during vancomycin/rifampicin combination treatment [51]. Thus, combining rifampin with another active antimicrobial for treatment of *Staphylococcus* sp. is necessary.

Another pharmacologic disadvantage is rifampin’s ability to act as a strong CYP450 and P-gp inducer. When combined with another antimicrobial, as is requisite for treating bacterial infections with rifampin, the standard antibiotic serum levels may be reduced. Thus, despite adding rifampin, the total antibactericidal activity for *S. aureus* may be decreased [47]. This potential has been demonstrated in vitro with oxacillin, nafcillin, vancomycin, and linezolid [52,53,54,55,56]. A method to detect the net effect of rifampin combinations on antibacterial activity is not established in the clinical setting.

### 3.2. Clinical Data with Rifampin

Based on current evidence, rifampin is not recommended as a routine adjunct in staphylococcal NVE [11]. Despite a prospective trial that showed no survival or time until blood culture clearance benefit with the addition of rifampin to vancomycin for IE caused by MRSA in the early 1990s, this practice was still observed, often for persistent positive blood cultures [57]. In a retrospective cohort analysis of *S. aureus* NVE IE comparing 42 cases (rifampin added to standard care) and 42 controls, there was no clinical benefit demonstrated with rifampin [47]. Cases were more likely to have a longer duration of bacteremia (5.2 vs. 2.1 days; *p* < 0.001), were less likely to survive (79% vs. 95%; *p* = 0.048), and trended toward higher rates of relapse (21% vs. 9%; *p* = 0.22). Cases received rifampin starting at a median of 3 days (range 0–19 days), median rifampin duration was 20 days (range, 14 to 48 days), and 16 of 42 patients (38%) were still bacteremic at the time rifampin was added. Rifampin resistance developed in nine cases where rifampin was received before bacteremia clearance (56%). Significant unrecognized drug–drug interactions with rifampin were frequent (52%) and consisted mainly of methadone, warfarin, and protease inhibitors. Drug-induced hepatitis also occurred in significantly more cases than controls (9 vs. 1; *p* = 0.014) but only in patients with hepatitis C virus (HCV) infection with mild baseline elevations of hepatic transaminases. Cases were also more likely to receive surgery (9 vs. 0; *p* = 0.03). While there was no clear benefit shown with the addition of rifampin, there are many notable limitations to these data. The authors rightly point out the rifampin cases may have been sicker at baseline, which prompted the addition of rifampin; this is also reflected in the surgical intervention disparity. The addition of rifampin during bacteremia may represent suboptimal timing, and the standard therapies used were also unbalanced.

In vitro studies have demonstrated that biofilm maturity can affect antimicrobial susceptibility within the biofilms, with younger biofilms being more susceptible to antimicrobials compared to mature biofilms [7]. Along the same lines, rifampin activity seems to depend on the timing of initiation with respect to bacterial inoculation in experimental studies, with earlier use and longer therapy demonstrating better efficacy compared to delayed initiation and shorter regimens [58,59]. However, active bacteremia may preclude early use in IE due to the risk of emergence of rifampin resistance due to the higher bacterial burden. Hence, rifampin initiation should not be “too early” when there is a higher bacterial burden with active bacteremia or undrained foci of infection, due to the increase in the risk of emergence of rifampin resistance, but not “too delayed”, where the theoretical benefit may be dampened by biofilm maturation. The perfect balance may be difficult to achieve in cases of prosthetic valve IE due to the unknown and unpredictable duration of bacteremia prior to and after the initiation of therapy; however, a longer duration of therapy initiated after the clearance of the bacteremia may be optimal until further clinical data are available from randomized controlled studies.

While the use of rifampin for staphylococcal NVE is generally only considered for those with CNS, bone, or joint involvement, supportive evidence has emerged specifically in patients with implants. In a cohort of patients with *S. aureus* bacteremia, the subgroup with implants had fewer late complications when treated with combination therapy (4.5% vs. 10.6%, *p* = 0.03, rifampin combination in 58.8%) suggesting a benefit of antibiofilm activity [60]. While no randomized prospective trials exist, the IDSA/AHA guideline recommended treatment for MRSA IE with PVE is vancomycin combined with rifampin for a minimum of 6 weeks, with 2 weeks of gentamicin with an evidence level B [11]. A recent meta-analysis sought to characterize the outcomes of staphylococcal PVE treated with adjunctive rifampin or gentamicin, or with both agents. Four studies were identified for inclusion, three of which are retrospective, and one a prospective registry study. Two studies (*n* = 201) suggested that adding rifampin to gentamicin-containing regimens did not reduce clinical failure (OR, 1.29 [95% CI, 0.71–2.33]) or infection relapse. One study found that rifampin use was associated with longer hospitalizations (mean, 31.3 vs. 42.3 days; *p* < 0.001). Safety data were not reported in all studies, but one study found rifampin was discontinued in 31% of patients due to hepatotoxicity, nephrotoxicity, or drug interactions [61].

### 3.3. Rifampin for IE Conclusions

While rifamycins hold a niche role in IE treatment, the drug interaction potential, toxicity profile, and survival evidence are a basis for careful risk–benefit clinical decision making. A future research direction is the use of rifabutin as an alternative to rifampin in IE, which has promising but limited case reports in orthopedic implant-associated infections and MRSA rat models of foreign body osteomyelitis [62,63]. Weighing the potential benefits demonstrated in vitro with the limited clinical evidence available (summarized in Table 2), an optimal scenario for rifampin use in IE is for patients that have no drug contraindications with staphylococcal PVE added to another active pharmacologic agent after blood cultures are negative.

## 4. Aminoglycoside Combination Therapy for Gram-Positive IE

Given the challenging nature of treating IE, it is not unexpected combination antimicrobials are recommended by the most recent IDSA/AHA guideline for IE treatment in many circumstances [11]. Combination antimicrobial therapy should be carefully considered in each patient; along with added antimicrobial exposure for the patient, there are added costs and adverse drug events [64,65]. Because of the narrow therapeutic index and lack of well-designed studies demonstrating clear clinical benefit to aminoglycosides for Gram-positive IE treatment combinations, their use remains controversial.

The use of aminoglycosides in IE has been hypothesized about and considered for combination therapy as far back as 1950, if not earlier [66]. The theory is that aminoglycosides can optimize bactericidal activity against high bacterial densities found within vegetations in IE, which can enable a shorter course of therapy in IE secondary to *Streptococcus* spp. [67]. Sexton et al. evaluated ceftriaxone 2 g once-daily for 4 weeks compared with ceftriaxone 2 g with gentamicin 3 mg/kg once-daily for 2 weeks on the outcome of clinical cure; there was no evidence of active endocarditis at the 3-month follow up. Clinical cure was nearly identical in the monotherapy (25/26 (96.2%)) and combination therapy (24/25 (96%)) groups. A major limitation was that only patients with NVE with penicillin-susceptible streptococci isolated were included; this is reflected by the IDSA/AHA recommendation of a 2-week course with NVE but a 4-week course maintained in the case of PVE [11].

While a shorter treatment duration for penicillin-susceptible streptococci NVE is established, the question remains whether an aminoglycoside should be added for IE with other pathogens. Drinkovic et al. evaluated IE secondary to staphylococcal species treated with monotherapy versus combination therapy with gentamicin [68]. There was an increased rate of negative valve cultures with combination therapy compared to monotherapy in patients with PVE, with no difference noted in NVE. While negative valve cultures may support aminoglycoside combination therapy, additional patient-focused outcome data are needed. Lastly, dual beta-lactam therapy with a gentamicin-containing combination IE therapy secondary to Enterococcus faecalis in 246 patients failed to show a difference in mortality (8% vs. 7%, *p* = 0.72), treatment failure (1% vs. 2%, *p* = 0.54), or relapse (3% vs. 4%, *p* = 0.67) [69].

However, though a potential benefit was shown in select clinical scenarios for aminoglycoside synergy, aminoglycoside use is not without risks. Nephrotoxicity risk, a hallmark of aminoglycosides, was demonstrated in a study comparing ampicillin with ceftriaxone to ampicillin with gentamicin in IE. There was an increase in acute renal failure with aminoglycoside-containing regimens (46% vs. 33%, *p* = 0.51) and an increased rate of discontinuation due to renal failure (23% vs. 0%, *p* < 0.001) [69]. Additionally, Cosgrove et al. evaluated different treatment regimens for IE and found that when low-dose gentamicin (3 mg/kg/d with renal adjustments) was used for a median of 5 days, there was a higher rate of renal dysfunction observed (22% vs. 8%, *p* = 0.005) [70]. Patients 65 years and older receiving gentamicin were independently associated with a clinically significant decrease in creatinine clearance. While shorter (2 week) regimens with gentamicin seem low risk, this study showed even a median treatment duration of 5 days (range 1–7) may have detrimental effects on renal function.

### Aminoglycoside Combination for IE Conclusions

The question remains: are the benefits of aminoglycoside-containing regimens in IE greater than the associated risks, mainly renal dysfunction? After a review of these data, as summarized in Table 3, it seems both monotherapy or non-aminoglycoside combination regimens are equally as efficacious as aminoglycoside-containing regimens in the treatment of IE caused by *Staphylococcus*, *Streptococcus*, or *Enterococcus* species. Given the risk of nephrotoxicity associated with any duration of aminoglycosides, their use should be reserved for cases not amenable to other treatment options (i.e., patients with a type 1 penicillin allergy or lack of culture clearance on preferred regimens).

## 5. Daptomycin and Ceftaroline Combination Therapy for Gram-Positive IE

The daptomycin (DAP) and ceftaroline (CPT) combination (CPT/DAP) is an emerging concept, leveraged for serious disease states including bacteremia and IE. There are multiple proposed theories for why these agents work synergistically together, including that CPT decreases cell wall thickness, increases cell wall fluidity, and decreases net surface charge, thus increasing DAP susceptibility [78].

In terms of the case reports detailing CPT/DAP for IE specifically, Cunha et al. describe a case when a patient with PVE secondary to MRSA experienced treatment failure following trials of vancomycin, linezolid, and quinupristin/dalfopristin prior to clinical improvement and negative cultures upon switching to CPT/DAP [71]. Similarly, Baxi et al. report a patient with NVE due to *S. aureus*; however, in this case the organism was deemed to be DAP non-susceptible and had intermediate vancomycin susceptibility [72]. On day 11 of therapy with persistent bacteremia, the patient’s course was adjusted to CPT/DAP, with negative blood cultures on day 21 and a diminished vegetation size 4 weeks after the end of therapy. Lastly, Sakoulas et al. detail Enterococcus faecalis IE refractory to gentamicin/ampicillin-sulbactam, ceftriaxone/ampicillin, and DAP/ampicillin, until negative blood cultures were demonstrated after switching to CPT/DAP [73]. Following this clinical success, the authors performed checkerboard assays on the isolated organism, displaying a decreased DAP MIC when utilized synergistically with CPT. While these are a few cases with positive clinical outcomes, it should be noted there are many cases where the outcome is not as successful, despite the use of CPT/DAP [79,80].

Fortunately, there are relatively robust data supporting CPT/DAP in patients with persistent bacteremia, oftentimes associated with IE. In a retrospective single-center review performed by Hornak et al., 10 patients (6 of whom had IE) were treated with CPT combination therapy after failure to clear blood cultures on vancomycin alone [74]. After a median bacteremia duration of 13 days (range 6–16), patients were switched to combination therapy with CPT, and 100% exhibited microbiologic cure in a median of 3 days (range 1–9), despite only two patients achieving adequate source control. While blood culture clearance was demonstrated with combination therapy, the smaller sample size and lack of comparison group prohibited the performance of statistical analyses. A meta-analysis of six trials evaluating either DAP or vancomycin monotherapy vs. combination therapy with CPT showed a statistically significant decrease in bacteremia recurrence with CPT combination therapy compared with monotherapy (OR = 2.95, 95% CI = 1.22–7.15, *p* = 0.02) but no difference in in-hospital mortality or adverse events [75]. Because CPT/DAP is resource intensive (cost and administration effort), it is of interest to use the combination for the shortest duration possible. A matched, retrospective, cohort study that included patients with MRSA bacteremia, with 53% having an endovascular source such as IE, compared CPT/DAP with vancomycin or DAP monotherapy and found a positive linear association between time before switch to combination therapy with clearance of blood cultures (r = 0.84, *p* < 0.001) [76]. In patients with an endovascular source, a numerically decreased incidence of mortality was associated with patients receiving CPT/DAP within 72 h of the initial positive blood culture compared with monotherapy (4.3% vs. 20.8%, *p* = 0.162), which supports the early use of CPT/DAP for IE.

The best time to stop combination therapy after the clearance of blood cultures has not been established. In the previously mentioned study by Hornak et al., CPT was stopped at discharge, which resulted in a wide range of combination therapy duration at 6–24 days (median 9) [74]. Nichols et al. assessed patients who received 10 or more days of CPT/DAP compared with patients receiving less than 10 days and required clearance of blood cultures and 72 h of combination therapy prior to de-escalating to monotherapy [56]. Of the patients included, there was a higher rate of IE in the group receiving at least 10 days of combination with CPT (56% vs. 35%, *p* = 0.01). In the primary composite outcome of bacteremia recurrence, infection-related mortality, or readmission, there was no difference found between treatment groups (21% vs. 24%, *p* = 0.66). Based on the results of this study, de-escalation may be considered after blood cultures are negative if the patient has been on CPT/DAP therapy for at least 72 h.

The safety profile of CPT has been studied in contexts outside of IE. The most common ADRs in a focused ADR study of CPT in 96 patients (15% had IE indication, 14% received DAP) were hematologic abnormalities, including leukopenia and neutropenia, with an overall ADR rate of 21% [81]. A previous study evaluating 527 patients found an ADR rate of 8%; however, the ADRs were not reported in detail, making it difficult to reach meaningful conclusions [82].

### Daptomycin and Ceftaroline Combination for IE Conclusions

While a mortality benefit has not consistently been shown with CPT combination therapy, a shortened time to negative blood cultures has been demonstrated. There is a paucity of data suggesting CPT/DAP as initial therapy in IE cases, and thus it is not universally recommended at this time, though earlier use may be better. Given the available data (summarized in Table 3), CPT/DAP should not be utilized as initial therapy; however it should be considered in the setting of treatment failure for staphylococcal or enterococcal IE.

## 6. Oral Antimicrobials for IE

With the recent interest in oral antibiotics for deep-seated infections, their role in IE is worth exploring. Standard IV therapy of 4–6 weeks can present several challenges, such as cost, prolonged hospital stay, medication-related adverse effects, IV access complications, or concerns for potential line misuse [83,84]. Additionally, the importance of concentration vs. route for bactericidal effects as well as the high serum concentration of modern antimicrobials support the exploration of oral options [85].

### 6.1. Current Guidelines

Clinical guidelines from expert organizations vary with recommendations for oral therapy for IE. Oral treatment with ciprofloxacin plus rifampin was studied as a potential option for uncomplicated right-sided MSSA in patients with IV drug use. However, adoption remained limited due to small sample size, lack of comparator arms, and decreasing susceptibility for ciprofloxacin [86,87]. Sulfamethoxazole/trimethoprim and clindamycin are also listed as alternatives for IE with *S. aureus* in the European Society of Cardiology guidelines but are not recommended by the IDSA/AHA guidelines. For resistant E. faecium, linezolid can be a good oral option due to its high bioavailability. Treatment is usually reserved for daptomycin non-susceptible strains due to linezolid inferiority, drug–drug interactions, and other adverse side effects. Lastly, the IDSA/AHA guidelines recommend ciprofloxacin for the HACEK group (Haemophilus aphrophilus, Actinobacillus actinomycetemcomitans, Cardiobacterium hominis, Eikenella corrodens, Kingella kingae) when cephalosporin intolerance is reported [11,88].

### 6.2. Efficacy of Oral Antibiotics as Stepdown Therapy for IE: Gram-Positive Organisms

The literature for primary use of oral antimicrobials for IE is mostly retrospective, observational, and with poor outcomes. The majority of the data exists for stepdown oral therapy after IV antimicrobials. Demonchy et al. conducted a retrospective audit of 66 IE cases to assess the quality of antimicrobial therapy. Antimicrobials were switched from IV to an oral route in 29% of patients (*n* = 19) with left-sided and/or complicated endocarditis, 18 ± 9 days after starting therapy, on average. Causative organisms were mostly Gram-positive, and there was no significant association between mortality and oral switch (0% in oral switch vs. 21% without, *p* = 0.052) [89]. A retrospective cohort of 426 cases over a period of 13 years evaluated oral stepdown therapy. Fifty percent of patients were switched to an oral agent after a median of 21 days. Antimicrobials included amoxicillin alone or in combination with clindamycin, fluoroquinolone, and/or rifampicin. Oral streptococci and *S. aureus* were predominant; however, patients in the oral group had fewer comorbidities, less severe disease, and less likelihood of infection with *S. aureus*. Switching to oral therapy was not associated with an increased risk of mortality, relapse, or reinfection [90].

Stamboulian et al. randomized 30 patients with penicillin-susceptible streptococcal IE to either 4 weeks of parenteral ceftriaxone (2 g once daily) or 2 weeks with ceftriaxone followed by 2 weeks of oral amoxicillin. All patients had left-sided endocarditis, uncomplicated cardiovascular history, and penicillin-susceptible streptococci strains. Therapeutic drug monitoring (TDM) was performed in almost all the patients, and serum concentrations displayed high bactericidal effects. A unique aspect of this study was that 27 of 30 patients were treated predominantly as outpatients, reducing hospital length of stay by 380 days, presumably in a system without routine OPAT for IE [84,91,92].

A quasi-experimental study compared IV therapy entirely (control) vs. oral stepdown after 1 week in patients with *S. aureus* endocarditis. Patients in the control group received oxacillin or vancomycin (MRSA) for 6 weeks plus once-daily IV gentamicin for 5 days. Patients in the stepdown group received IV co-trimoxazole with IV clindamycin for 7 days, then 5 weeks of oral co-trimoxazole alone. Seven days of IV rifampicin and gentamicin were added in case of persistence. As expected, average length of hospital stay was significantly less in the oral group (difference of 10 days, *p* = 0.005). Patients in the oral group were less sick and had fewer complications, lower in-hospital mortality, and lower 30-day mortality. There were no significant differences between 90-day mortality and relapses between both groups. Almost 29% of patients were additionally treated with gentamicin and rifampin, making it difficult to declare causality. As a rare study showing mortality benefit, this offers promising results for oral options [84,93].

In terms of oral antibiotics for IE, the POET trial is the most recent and the largest randomized-controlled trial. This multicenter, unblinded, non-inferiority study evaluated oral stepdown treatment for left-sided endocarditis caused by *S. aureus*, coagulase-negative *Staphylococcus* spp. (CoNS), streptococci, or Enterococcus faecalis. A transesophageal echocardiogram (TEE) was conducted pre-randomization, and at the end of treatment for all patients, and participants were followed 6 months after treatment completion. All 400 patients completed at least 10 days of IV antibiotics and were then randomized to continue on an IV regimen or switch to oral antimicrobials. Oral regimens were unique to the study and consisted of two different classes of drugs. Approximately half of the patients had aortic valve endocarditis, and one-third had mitral valve endocarditis. Streptococci were the most common pathogens, followed by *S. aureus*, *E. faecalis*, and CoNS. The primary endpoint of treatment failure was a composite of all-cause mortality, unplanned cardiac surgery, embolic events, and relapse of bacteremia with primary pathogen. At 6 months, treatment failure had occurred in 12.1% of patients in the IV group vs. 9% in the oral group, meeting the threshold for non-inferiority. All-cause mortality and safety outcomes were similar between the two groups [94]. A 5-year follow-up published in 2022 showed that the primary composite outcomes had occurred in 32.8% of patients in the stepdown group and in 45.2% of the IV group, mainly driven by all-cause mortality [95]. Strengths of the study include inclusion of stable patients with endocarditis and those requiring surgery, large sample size, and great adherence to regimens. Some factors that make this study non-generalizable include lack of MRSA IE, small percentage of patients with IV drug use, only left-sided endocarditis, unknown IV regimens, high doses of oral antibiotics, need for therapeutic drug monitoring, and the high follow-up rate [84,94,95]. However, this landmark trial has paved the way for exploring oral stepdown therapy further.

Freling et al. utilized published literature and national guidance to establish a set of expectations for clinicians. The aim was to achieve more standardized, equitable care based on real-life practice conditions within their facility. Unlike previous studies, patients could be transitioned to oral therapy if they were clinically stable without need for surgical intervention, cleared blood cultures with initial IV course, had no concerns regarding enteral absorption, did not have psychosocial concerns affecting compliance, and the organism was susceptible to the oral regimen selected. The primary efficacy endpoint of this retrospective review was clinical success, defined as being alive, without recurrent bacteremia, and without treatment-emergent infectious complications within 90 days. This study had a significantly higher proportion of tricuspid-valve endocarditis, patients with history of injection drug use (IDU), and MRSA cases in the oral arm than the previously published literature (20.4% IV only vs. 34.8% oral therapy; *p* = 0.004). Clinical success rates were similar in both the IV-only arm and the oral therapy arm at 90 days of follow-up (84.4% vs. 87%, *p* = 0.66) and at last follow-up (82.0% vs. 76.1%, *p* = 0.36). The IV-only group had significantly more adverse events (8.7% vs. 27.5%; *p* = 0.004), with acute kidney injury (AKI) and line-related causes being most notable. Linezolid with or without rifampin, followed by high-dose penicillins, were the most common oral agents utilized. Both arms had demographic differences that could impact the outcomes, such as older age, higher rates of diabetes, ESRD, and left-sided disease in the IV-only group, and higher rates of substance use disorders and MRSA infections in the oral stepdown group. However, a multivariable logistic regression failed to demonstrate any significant impact on the primary endpoint. Although retrospective, this study further offers local evidence for use of oral antibiotics in deep-seated infections with rational clinical criteria [96].

Availability and dosing of oral antimicrobials, especially in Gram-positive IE, is highly variable in the available literature. Past studies have included TDM as part of their protocol, which is not ubiquitously available. Thus, a detailed review of PK and PD principles of oral options is outside the scope of this manuscript.

### 6.3. Oral Antibiotics in Injection Drug Use (IDU)

The majority of IE studies focus on left-sided endocarditis. Between 2000 and 2013, IE-related hospitalizations from IDU increased from 7% to 12% [97]. With concerns for central line misuse, non-compliance, and healthcare resource burden, this population may be well suited for oral antibiotic regimens. In 1980, Parker and Fossieck described their experience with oral stepdown therapy in *S. aureus* IE. With a majority IDU population, patients received a mean of 16.4 days of IV therapy before switching to oral options. Therapeutic drug monitoring was conducted while on IV and oral treatment, and doses were adjusted accordingly. Serum drug levels were similar in both groups, and all patients achieved microbiological and clinical cure at the 6-month follow-up [98]. A 1989 study enrolled 14 patients with right-sided *S. aureus* IE secondary to IDU. Patients were treated with 1 week of IV ciprofloxacin and then switched to oral ciprofloxacin for 3 weeks; all received 28 days of rifampicin. Ten of 14 completed therapy and were determined to have microbiological and clinical cure, despite low peak:MIC ratios [86]. Heldman et al. randomized patients with IDU to either oral (ciprofloxacin and rifampicin) or IV (oxacillin or vancomycin plus 5 days of gentamicin) at presentation. *S. aureus* was isolated in 94% of the included patients. There was no statistically significant difference in treatment outcome between the groups, but the IV group had a higher incidence of toxicity (61.5% vs. 2.8%, *p* < 0.0001) [87].

Some of the previously discussed studies also included patients with IDU. This population accounted for 12.9% of the co-trimoxazole plus clindamycin group and 15.3% of the control group in a 2019 study, which was an appreciable representation [93]. Conversely, the scarce IDU population in the POET trial limits its generalizability (1.5% in the IV group and 1% in the oral group) [94]. The recent study by Freling et al. included a good representation of patients with IDU (18% in the IV group vs. 37% in the oral group) and MRSA but still showed no difference in the clinical outcomes [96]. Further research in right-sided IE, specifically in MRSA, and with consistent dosing of oral antimicrobials can revolutionize IE treatment options in this high-risk population.

### 6.4. Dual Therapy for Non-HAEK Gram-Negative IE

Although Gram-negative bacilli are uncommon causes of endocarditis, non-HACEK organisms can be implicated in patients with nosocomial exposures, prosthetic valves, intravascular catheters, intravenous drug use, or immunocompromising conditions [99,100]. High mortality and limited evidence are reasons that current guidelines recommend combination therapy with a beta-lactam and either an aminoglycoside or fluoroquinolone for 6 weeks [11]. Comparative studies for efficacy and safety with monotherapy vs. combination therapy are lacking. Morpeth et al. organized a prospective, multinational cohort database to describe the clinical characteristics and outcomes of patients with non-HACEK gram-negative endocarditis. Thirty-eight percent of patients received monotherapy with a β-lactam, aminoglycoside, or a fluoroquinolone. Sixty-three percent of patients received combination therapy with a β-lactam plus an aminoglycoside, β-lactam plus a fluoroquinolone, or all three of the agents. There was no significant difference in mortality rate between patients who received monotherapy or combination therapy (4 of 18 patients (22%) vs. 8 of 30 patients (27%), respectively; *p* = 0.73) [99]. Since then, only one other study has performed this comparison. Lorenz et al. evaluated a composite of 60-day all-cause mortality, readmission, or recurrence of bacteremia in 60 patients diagnosed with non-HACEK IE who received combination therapy or monotherapy. The primary composite outcome occurred in 57% of patients, but there was no difference between the groups (62% monotherapy vs. 50% combination therapy; *p* = 0.36). There were no significant differences in inpatient mortality, infectious embolic events, or the multivariable logistic regression model for proven confounders. Time to culture clearance and median length of stay was longer in the combination therapy group (3 vs. 2 days, *p* = 0.003; 20.5 vs. 12 days, *p* = 0.003 respectively), but this could be attributed to a more severe disease process. Five patients in the aminoglycoside combination group experienced acute kidney injury compared to none in the monotherapy group (*p* = 0.012). In spite of the small sample size and retrospective nature, this study provides an avenue to explore MT in patients with uncomplicated, non-HACEK, Gram-negative IE [101].

### 6.5. Future Studies

The RODEO trials are multicenter, randomized, open-label trials that will compare oral switch vs. IV antibiotic therapy in patients with left-sided IE. RODEO-1 will assess non-inferiority outcomes in left-sided IE with staphylococci, and RODEO-2 will be dedicated to streptococci and enterococci. The design is similar to the POET trial but also includes secondary outcomes like patient quality of life, echocardiographic outcome, and the costs and efficiency associated with IE care [102]. Another randomized open-label study aims to compare IV continuation vs. oral switch in PWID. With important outcomes like 90-day mortality and compliance, this non-inferiority trial has great potential to advance care in this high-risk population [103].

### 6.6. Oral Antimicrobials in IE Conclusions

There is much interest and enthusiasm for oral antimicrobials in IE. Important factors such as Gram-positive vs. Gram-negative organisms, secondary complications, risk factors for compliance, and PK/PD parameters need to be considered when utilizing these agents. Oral antimicrobials for IE caused by Gram-positive bacteria remain as stepdown options, and studies lack outcome data for MRSA (evidence summarized in Table 4). Future studies targeting oral agents in MRSA, especially in PWID, will offer significant reprieve in transitions of care for these patients. Current guidelines recommend dual therapy for uncomplicated, non-HACEK IE, but the risk of ADEs often outweighs the benefit. Prospective studies, similar to the RODEO trials, for MT in Gram-negative IE will help provide further insight and support.

## 7. Long-Acting Lipoglycopeptide Antibiotics for IE

Before their formal approval in 2014 for acute bacterial skin and skin structure infections (ABSSSIs), there was interest in long-acting lipoglycopeptide antibiotics (LALAs), dalbavancin (DAL) and oritavancin (ORI), to play a role in complicated and multi-drug-resistant infections. A foreign-body guinea pig infection model from 2013 demonstrated that DAL prevented the emergence of rifampin resistance within biofilms [104]. Shortly thereafter, a human case report described successful use of ORI, obtained via compassionate use prior to market availability, for a highly complex case of vancomycin-resistant Enterococcus faecium PVE [105]. It is clear that there remains great interest in this area.

Clinical data specific to LALAs in IE are currently limited to case series and retrospective reviews; the only randomized controlled trials utilizing LALAs are for ABSSSIs. Given the nature of retrospective and small-scale studies, external validity has been called into question for existing data. Specific areas of heterogeneity amongst LALA IE studies include inconsistent reporting of confounding factors (e.g., surgical source control and duration of prior antibiotics), varying definitions of “cure” and/or “failure”, and a wide range of dosage regimens. IE is not a simple disease state to study in general, and LALAs add an additional layer of complexity.

Due to limited availability of commercial susceptibility testing for both DAL and ORI, clinicians often rely on surrogate routinely tested antibiotics, such as vancomycin. While DAL and ORI are often considered to be clinically similar, differences in mechanism of action have the potential for clinical implications. DAL exhibits time-dependent bactericidal activity and inhibits cell wall synthesis via a similar mechanism to vancomycin, whereas ORI exhibits rapid concentration-dependent bactericidal activity via cell wall synthesis inhibition and perturbation of membrane barrier function. An in vitro time-kill study found that ORI exerts bactericidal activity against MRSA in a nondividing state, whereas the antibacterial activity of DAL was diminished [106]. More studies are needed to assess if these differences are clinically relevant.

### 7.1. Dalbavancin Evidence

The majority of data describing outcomes in IE utilize DAL. While the driver for this selection is not specified in studies, the use of DAL may reflect both institutional formulary preferences and wider international availability. Of the four DAL studies including 19 or more patients with IE, clinical cure rates ranged from 72.2% to 100%, with all but one reporting success rates above 90% [107,108,109,110].

The largest dataset exclusively reporting outcomes of DAL for treatment of IE yielded a clinical and microbiological success rate of 92.6% (25/27) [107]. Of the 27 patients included, 15 had NVE, 7 had PVE (1 was suspected), and 5 had cardiac-device IE. *Staphylococcus aureus* was the primary pathogen in NVE (N = 6), streptococci in PVE (N = 4), and coagulase negative staphylococci in cardiac devices (N = 3). Other pathogens included Enterococcus faecalis and *Aerococcus* spp. Surgical source control was obtained in 11, 1, and 4 patients with NVE, PVE, and cardiac device IE, respectively. The majority (88.9%) of patients received other antimicrobials for 1 to 6 weeks prior to DAL. The median duration of DAL was 6 weeks (range 1 to 30), and all patients received at least 1 dose of DAL in the hospital prior to discharge. Most patients (63%) received DAL 1500 mg for their first dose, followed by 1000 mg every other week thereafter; the remaining patients received DAL 1000 mg for their first dose, followed by 500 mg once weekly thereafter. Of the two failures, one patient passed away two weeks after prosthetic valve replacement surgery due to postoperative complications. The second patient, with a cardiac device infection, had incomplete surgical source control; clinical and microbiological failure were declared when blood cultures were positive on day 210 of a 30-week course of DAL. Based on the results from this study, DAL may be an effective therapy for patients after at least one week of other antibiotics.

Another large DAL study included patients with IE and/or Gram-positive bacteremia [108]. Of the 34 patients with IE, 11 had NVE, 15 had PVE, and 8 involved pacemaker leads. The most commonly involved valve was aortic (50%), followed by mitral (23.5%) and tricuspid (2.9%). Of those with IE, clinical cure was 100%, microbiological cure was 97.1%; no deaths or relapses were reported through 12 months. The most commonly implicated organisms were CoNS (42.9%), followed by MSSA (20%), streptococci (20%), MRSA (8.6%), and *E. faecalis* (8.6%). Surgery was performed in 34.3% of cases, and antibiotics were administered for a median of 28 days prior to DAL. At least eight different dosing strategies were utilized. The most common, used in 29.4% of cases, was 1000 mg on day one followed by 500 mg on day eight; patients were covered for a median of 14 days with DAL. The results of this study suggest that DAL may be an effective therapy to complete the final 2 weeks of a 6-week regimen, regardless of which valve is involved or if surgical source control was obtained.

The optimal dosing regimen for DAL is difficult to define, as studies utilized numerous variations of dose, frequency, and total doses administered. Regimens should take into account prior antimicrobial exposure to determine how many doses of DAL may be needed to complete treatment. As there is a lack of consistency between studies with regard to dose and frequency, a conservative approach is recommended until further studies better define optimal regimens. A reasonable approach would be an initial dose of 1500 mg followed by 500 mg to 1000 mg once weekly to complete the intended number of total weeks of therapy. Given that DAL was rarely utilized as primary therapy, it is unclear if DAL should be considered over SOC for initial treatment.

Although there are currently insufficient data in IE, future approaches to dosing DAL may incorporate TDM [111]. A proof-of-concept TDM study in osteoarticular infections found that the median effective DAL concentration is expected to drop below the target threshold after 5 weeks when two 1500 mg DAL doses are administered 1 week apart. The authors noted interpatient PK variability, particularly in patients under the fifth percentile, which may lead to lower concentrations before 5 weeks. Of note, this study utilized a MIC breakpoint of 0.125 mcg/mL for *S. aureus*, whereas the Clinical Laboratory Standards Institute’s (CLSI) susceptibility breakpoint is 0.25 mcg/mL [112]. Ultimately, reliance on population-based kinetics may unintentionally promote prolonged subtherapeutic concentrations, which may potentiate the development of resistance while on therapy—a trend already emerging in the lab and in practice. In an in vitro study, DAL exposure selected for DAL-non-susceptible and vancomycin-intermediate strains of MRSA, with an eightfold increase in DAL MIC as early as day four [113]. At least two clinical cases demonstrated this is a valid concern in clinical practice. In one report, a vancomycin-intermediate strain of *S. aureus* emerged after treatment with DAL for an MRSA central line–associated bloodstream infection [114]. In a second case, a patient returned with DAL-, vancomycin-, and daptomycin-nonsusceptible MRSA IE, just 5 weeks after treatment with two doses of DAL for an MRSA arteriovenous fistula infection [115]. Given the current evidence, further investigations into both TDM and monitoring for emergent antimicrobial resistance are warranted.

### 7.2. Oritavancin Evidence

Outcomes with ORI for IE are limited to four patients in three studies [105,116,117]. These cases highlight the difficulty in studying a cohesive patient population due to considerable differences. In each of these studies, patient characteristics, dosage regimens, and implicated organisms varied significantly. Ahiskali and Rhodes included two cases of NVE amongst a larger cohort of PWID and/or those who were experiencing homelessness. In both cases, over 4 weeks of other antimicrobials were administered prior to ORI, and surgical source control was not achieved. The patient with MSSA IE was deemed a clinical success, whereas the patient with MRSA and concomitant vertebral osteomyelitis was deemed a failure [116]. Stewart and colleagues described a patient with Group B Streptococcus NVE included in their larger dataset. The patient received 7 days of antimicrobials prior to a single dose of ORI 1200 mg and was deemed as treatment failure as the patient required valve replacement within 3 months of the index encounter [117]. Finally, Johnson and colleagues described a markedly complicated case of VRE prosthetic valve IE that was treated with >10 doses of ORI over an extended period of time, along with other antibiotics. Treatment failures occurred at various points during the prolonged course; however, it was ultimately deemed a success [105].

Similarly to DAL, the optimal dosage regimen for ORI in the treatment of IE is unclear. If a shorter lead-in period of IV antimicrobials is used (e.g., a week or less), more than one dose of ORI should be administered to patients. A conservative dosing regimen would be 1200 mg followed by 800 mg once weekly thereafter for the number of weeks needed for the full course.

Currently there are no data regarding use of TDM-guided ORI dosing for any infection. Similarly, there are no reports describing the emergence of resistance to ORI while on therapy. Although ORI has three mechanisms of action that may provide it a higher barrier to resistance, conclusions cannot be drawn regarding antimicrobial resistance until more data become available.

### 7.3. LALA in IE Conclusions

Based on limited available data (summarized in Table 5), LALAs may be an effective option for treatment of IE after completion of at least 1 week of traditional IV antibiotics. The majority of current studies utilize DAL; however, ORI may be a reasonable alternative based on its similar pharmacokinetic and pharmacodynamic profile. More studies are needed to define their place in therapy, optimal dosing regimens, and risk of developing antimicrobial resistance with prolonged exposure.

## 8. Conclusions

Despite decades of research, there are standing debates and new questions surrounding IE treatment. We have summarized the relevant evidence and provided our interpretation of its implications (Table 6). The optimal agent for MSSA IE is debatable, with both ASPs and cefazolin as viable options. Rifampin has a niche role for staphylococcal PVE due to unique biofilm activity in patients that do not have contraindications due to drug interactions. Ceftaroline combinations are emerging for persistent blood culture positive IE, while aminoglycoside combinations continue to be beleaguered by toxicity risk. Stepdown therapy to oral antibiotics can be considered in stable patients after a period of IV antimicrobial therapy, with emerging studies to assess use in high-risk patients. Lastly, long-acting lipoglycopeptide antibiotics are becoming established as alternatives to overcome common logistical barriers in IE treatment.

## Figures and Tables

**Table 1:** Clinical Evidence Summary for Beta lactam selection for Methicillin-Susceptible *Staphylococcus aureus* Endocarditis.

Citation	Study Design and Methods	Notable Outcomes	Conclusion
Open Forum Infect Dis. 2019, 6(7), ofz270. [18]	Retrospective cohort study of patients with MSSA bacteremia treated exclusively with nafcillin, oxacillin, cefazolin, piperacillin/tazobactam or fluoroquinolones	Similar 30-day mortality between ASPs and cefazolin (HR 0.67, 95% CI 0.11–4.00) Lower 30-day mortality for ASPs/cefazolin compared to piperacillin/tazobactam when propensity matched (HR 0.10, 95% CI 0.01–0.78)	Piperacillin/tazobactam should not be used preferentially in patients with MSSA bacteremia
Clin Microbiol Infect. 2011, 17(10), 1581–6. [19]	Retrospective cohort study of 30-day and 90-day mortality in patients with MSSA bacteremia managed with beta-lactam therapy	30-day mortality significantly higher in patients receiving ceftriaxone (OR 2.24, 95% CI 1.23–4.08) or BL/BLI (OR 2.68, 95% 1.23–5.85) No difference in 90-day mortality between cloxacillin vs. cefazolin treated patients (HR 0.91, 95% CI 0.47–1.77)	BL/BLI and ceftriaxone should not be used as preferential therapies in patients with MSSA bacteremia
Open Forum Infect Dis. 2022, 9(Suppl 2), ofac492.029. [20]	Retrospective cohort study of clinical outcomes of MSSA bacteremia managed with ceftriaxone vs. cefazolin or ASP	Higher rate of 30-day and 90-day treatment failure in patients treated with ceftriaxone as compared to those treated with cefazolin or ASPs (3.8% and 8.6% vs. 8.1% and 27%)	Cefazolin or ASPs should be used preferentially over ceftriaxone for MSSA bacteremia
Open Forum Infect Dis. 2018, 18(5), ofy089. [21]	Retrospective cohort study of patients with MSSA bacteremia managed definitively with ≥14 days of either cefazolin or ceftriaxone	Higher rate of therapy extension, incomplete therapy, unplanned oral suppression, relapse, or hospital admission or surgery within 90 days observed in patients receiving ceftriaxone as compared to cefazolin (54.5% vs. 28.9%, *p* = 0.29)	Cefazolin should be used preferentially over ceftriaxone in the treatment of MSSA bacteremia
Int J Antimicrob Agents. 2022, 60(3), 106632. [22]	Retrospective cohort study comparing safety and efficacy of cefazolin and ceftriaxone in MSSA bacteremia	Similar rates of clinical cure at 28 days or hospital discharge between cefazolin and ceftriaxone treated patients (90.1% vs. 86.2%, *p* = 0.359).	Ceftriaxone may represent a viable alternative to cefazolin in the management of MSSA bacteremia
Open Forum Infect Dis. 2020, 13(7), ofaa341. [23]	Retrospective cohort study evaluating patients with MSSA bacteremia receiving ≥7 days of cefazolin or oxacillin vs. ceftriaxone as OPAT	No differences in microbiologic failure, 90-day mortality, or readmission between cefazolin or oxacillin vs. ceftriaxone for MSSA bacteremia (19% vs. 21%, *p* = 0.7)	Ceftriaxone may represent an alternative to cefazolin or ASPs for patients with MSSA bacteremia being managed in the outpatient setting
Antibiotics. 2022, 11(3), 375. [24]	Systematic review and meta-analysis evaluating outcomes of MSSA BSIs treated with ceftriaxone as compared to SOC	No difference noted between ceftriaxone and SOC with regards to clinical cure (OR 0.65, 95% CI 0.29–1.45) or microbiological cure (OR 1.48, 95% CI 0.29–7.51)	Ceftriaxone represents an appropriate alternative to standard of care for patients receiving therapy for MSSA BSI
Microb Drug Resist. 2014, 20(6), 568–74. [28]	Retrospective cohort study evaluating outcomes of patients with MSSA bacteremia managed with cefazolin stratified by presence of CzIE	CzIE was associated with higher rates of persistent bacteremia (9% vs. 0%, *p* = 0.04) but was not associated with a statistically significant increase in treatment failure (48% CzIE vs. 25% no CzIE, *p* = 0.13) Site of infection, but not CzIE was associated with treatment failure on multivariable analysis	CzIE may contribute to bacteremia persistence, but was not found to impact clinical outcomes
Open Forum Infect Dis. 2018, 5(6), ofy123. [29]	Prospective study evaluating the impact of CzIE on 30-day mortality in patients with MSSA bacteremia treated with cefazolin	CzIE was found to be associated with increases in 30-day mortality in both univariate (*p* = 0.034) and multivariable analysis (*p* = 0.03)	Cefazolin should be used with caution as first line therapy in patients with MSSA bacteremia shown to be positive for CzIE
Clin Infect Dis. 2017, 65(1), 100–106. [30]	Retrospective cohort study evaluating clinical outcomes of patients with MSSA bacteremia managed definitively with cefazolin vs. ASPs	Patients receiving cefazolin demonstrated lower rates of 30-day (HR 0.63, 95% CI 0.51–0.78) and 90-day (HR 0.77, 95% CI 0.66–0.90) mortality compared to ASPs.	Cefazolin may be more effective than ASPs in the management of MSSA bacteremia
BMC Infect Dis. 2018, 18, 508. [31]	Systematic review and meta-analysis of safety and efficacy of cefazolin vs. ASPs in the management of MSSA bacteremia	Cefazolin was associated with lower rates of mortality (OR 0.69, 95% CI 0.58–0.82), clinical failure (OR 0.56, 95% CI 0.37–0.85) as compared to ASPs with no difference in recurrence (OR 1.12, 95% CI 0.94–1.34) Cefazolin was associated with a lower rate of discontinuation due to ADE compared to ASPs (OR 0.24, 95% CI 0.12–0.48)	Cefazolin should be favored over ASPs when making definitive antimicrobial selections for patients with MSSA bacteremia
Infect Dis Ther. 2019, 8(4), 671–686. [32]	Meta-analysis with trial sequential analysis evaluating differences in outcomes between patients with MSSA bacteremia managed with cefazolin vs. those managed with ASPs	Cefazolin, as compared to ASPs, was associated with lower all-cause mortality (*p* < 0.01), clinical failure (*p* < 0.01), and antibiotic discontinuation due to ADEs (*p* < 0.01) Cefazolin associated with a higher rate of infection recurrence (OR 1.41, *p* = 0.03)	Cefazolin is a reasonable first line beta-lactam for use in the management of patients with MSSA bacteremia
J Antimicrobial Chemother. 2018, 73(10), 2643–2651. [33]	Systematic review and meta-analysis evaluating differences in clinical outcomes between patients managed with cefazolin and those managed with ASPs	Cefazolin associated with lower rates of 90-day mortality (OR 0.63, 95% CI 0.41–0.99) and discontinuation due to ADE (OR 0.25, 95% CI 0.11–0.56) Cefazolin and ASPs demonstrated similar rates of clinical failure (OR 0.85, 95% CI 0.41–1.76)	Cefazolin, in addition to ASPs, may represent an important first-line agent in the management of MSSA bacteremia
Clin Microbiol Infect. 2021, 27(7), 1015–1021. [34]	Retrospective cohort study evaluating outcomes of patients with MSSA IE managed definitively with either cefazolin or ASPs	No difference in 90-day mortality observed between cefazolin or ASPs (24.5% vs. 28.7%, *p* = 0.561) Premature antimicrobial discontinuation due to ADE was more commonly seen in patients receiving ASPs compared to those receiving cefazolin (0% vs. 8.2%, *p* = 0.042)	Cefazolin is an appropriate alternative to ASPs as a definitive agent in the management of MSSA IE
Antimicrob Agents Chemother. 2016, 60(5), 3090–3095. [35]	Retrospective cohort study comparing tolerability differences between oxacillin and nafcillin	Oxacillin was associated with significantly less hypokalemia (17% vs. 51%, *p* < 0.01) and less premature discontinuation due to ADEs (2% vs. 18%, *p* < 0.01) than nafcillin	Oxacillin is better tolerated than nafcillin

**Table 2:** Clinical Evidence Summary for Rifampin for Staphylococcal Infective Endocarditis.

Citation	Study Design and Methods	Notable Outcomes	Conclusion
Rev Infect Dis 1983; 5(Suppl 3):S543–8. [52]	Retrospective cohort study assessing the role of rifampin in patients treated with vancomycin or a beta-lactam for MRSE PVE	13/15 (87%) of patients receiving rifampin and VAN were considered cured and an increase in serum bactericidal activity was observed. 7/15 of these patients also received an AG 3/8 (38%) of patients receiving rifampin and beta-lactam were cured (87% vs. 28%, *p* = 0.025). 1/8 of these patients also received AG Two rifampin-resistant strains were isolated from surgical cultures	Cure rates obtained with rifampin plus vancomycin (with or without an aminoglycoside) were encouraging for further study Beta-lactam (with or without rifampin) should not be used to treat MRSE PVE
Ann Intern Med 1991; 98:447–55. [57]	Retrospective cohort study assessing the role of rifampin in patients treated with vancomycin or a beta-lactam for MRSE PVE	Failure rate (composite of death and recurrence in up to 3 months) occurred in 5/10 (50%) in those receiving VAN or beta-lactam alone 23/46 (50%) of patients received rifampin and 21/46 (45.7%) received AG in addition to VAN or a beta-lactam Failure occurred in 5/15 (33.3%) of patients receiving rifampin combination and in 2/8 (25%) of patients receiving rifampin and AG combination	Failure rates were lower in cohorts of patients receiving combinations that included rifampin; however, sample sizes were very small and use of beta-lactam for MRSE is no longer considered standard care
Clin Infect Dis 2021; 72:e249–55. [61]	Observational retrospective cohort study of adults with staphylococcal PVE at 3 referral centers	Staphylococcus aureus (63.3%) and coagulase-negative staphylococci (36.7%) were included, MRSA was associated with one year mortality 101/180 (56.1%) patients were treated with rifampin (median duration of 33.0 days) and 79/180 (43.9%) patients had no rifampin One-year mortality was 38/101 (37.6%) in patients treated with rifampin and 25/79 (31.6%) in those without rifampin (*p* = 0.62) Relapse rates occurred in 6/101 (5.9%) with rifampin and 7/79 (8.9%) in those without (*p* = 0.65) Patients treated with rifampin had longer hospital length-of-stay.	One-year survival and relapse rates were not statistically different in patients treated with or without rifampin. Relapse rate was numerically lower in those treated with rifampin combination

Abbreviations—AG: aminoglycoside; PVE: prosthetic valve endocarditis; MRSA: methicillin-resistant *Staphylococcus aureus*; MRSE: methicillin resistant *Staphylococcus epidermidis*; VAN: vancomycin.

**Table 3:** Clinical Evidence Summary for Combination Antimicrobials for Gram Positive Endocarditis.

Citation	Study Design and Methods	Notable Outcomes	Conclusion
*Aminoglycoside Combination*			
Clin Infect Dis. 1998, 27(6), 1470–1474. [67]	Randomized controlled trial evaluating ceftriaxone monotherapy × 4 weeks vs. Ceftriaxone/AG × 2 weeks in streptococcal NVE	No difference in clinical cure rate (96.2% vs. 96%)	A shorter duration of therapy can be considered with the addition of gentamicin to treat streptococcal NVE
J Antimicrob Chemother. 2003, 52(5), 820–825. [68]	Randomized controlled trial evaluating SOC monotherapy vs. SOC/gentamicin for staphylococcal IE	Increased rate of negative valve culture with combination therapy in PVE	PVE due to staphylococcal species benefit from combination therapy compared with monotherapy for culture clearance
Clin Infect Dis. 2013, 56(9), 1261–1268. [69]	Randomized controlled trial evaluating dual beta-lactam vs. gentamicin-containing combination therapy for enterococcal IE	No difference in mortality. Increased risk of renal failure with aminoglycoside regimen	Dual beta-lactam containing regimens are equally efficacious as a gentamicin-containing regimen for enterococcal IE and have shown decreased nephrotoxicity
Clin Infect Dis. 2009, 48(6), 713–721. [70]	Various treatment regimens evaluated	Increased rate of renal toxicity with gentamicin containing regimens (22% vs. 8%)	When used for a median of 5 days, nephrotoxicity is demonstrated in aminoglycoside-containing regimens
*Ceftaroline/Daptomycin Combination*			
Int J Antimicrob Agents. 2015, 46(2), 225–226. [71]	Patient case of MRSA IE treatment failure to 3 regimens prior to switching to CPT/DAP	Clearance of blood cultures observed upon switch to CPT/DAP	CPT/DAP can be considered as rescue therapy for blood culture clearance in IE secondary to MRSA
Infection. 2015, 43(6), 751–754. [72]	Patient case describing a DAP non-susceptible, VAN intermediate *S. aureus* IE with persistent bacteremia transitioned to CPT/DAP	Negative blood cultures and diminished vegetation size observed upon CPT/DAP initiation	CPT/DAP is a viable treatment option for persistent bacteremia despite DAP non-susceptibility
Antimicrob Agents Chemother. 2013, 57(8), 4042–4045. [73]	Patient case of enterococcal IE treatment failure to 3 regimens prior to switching to CPT/DAP	Clearance of blood cultures observed upon switch to CPT/DAP	CPT/DAP can be considered as rescue therapy for blood culture clearance in IE secondary to *E. faecalis*
Ther Adv Infect Dis. 2019, 6, 2049936119886504. [74]	Series of case reports including 10 patients (6 with IE) were switched to CPT/DAP after vancomycin failure for bacteremia treatment	Median 13 days of persistent bacteremia prior to switch and median 3 days of blood culture clearance following switch	CPT/DAP has shown a decrease in blood culture clearance following vancomycin failure in persistent bacteremia (60% of patients with IE)
Antibiotics (Basel). 2022, 11(8), 1104. [75]	Meta-analysis including 6 trials comparing VAN or DAP monotherapy vs. CPT/DAP or CPT/VAN combination therapy	Decrease in bacteremia recurrence with CPT combination. No difference in in-hospital mortality.	Combination therapy with CPT has a beneficial effect on bacteremia recurrence, however no difference shown in mortality vs. monotherapy
Open Forum Infect Dis. 2019, 7(1), ofz538. [76]	Matched, retrospective cohort evaluating MRSA bacteremia therapy with monotherapy or CPT combination therapy	Positive linear association found between time before switch to CPT combination and time to blood culture clearance (r = 0.84)	The earlier therapy is adjusted to include CPT in the treatment of MRSA bacteremia, the earlier time found to blood culture clearance
Open Forum Infect Dis. 2021, 8(7), ofab327. [77]	Retrospective study evaluating patients maintained on CPT combination therapy vs. de-escalation to monotherapy without CPT	No difference found in bacteremia recurrence, mortality, or readmission between patients receiving >10 days vs. <10 days of CPT combination	De-escalation from CPT combination therapy with less than 10 days total duration had similar outcomes compared with longer durations

Abbreviations—AG: aminoglycoside; CPT: combination therapy; DAP: daptomycin; IE: infective endocarditis; MRSA: methicillin-resistant *Staphylococcus aureus*; NVE: native valve endocarditis; PVE: prosthetic valve endocarditis; SOC: standard of care; VAN: vancomycin.

**Table 4:** Clinical Evidence Summary for Oral Antimicrobials.

Citation	Study Design and Methods	Notable Outcomes	Conclusion
*Step-down therapy in Gram-positive IE*			
Int J Antimicrob Agents. 2019 Aug;54(2):143–148. doi: 10.1016/j.ijantimicag.2019.06.006. Epub 2019 Jun 8. PMID: 31181351. [93]	Quasi-experimental study Control: oxacillin or vancomycin IV × 6 weeks PLUS once-daily IV gentamicin × 5 days Oral step-down: IV co-trimoxazole with IV clindamycin for 7 days, then 5 weeks of oral co-trimoxazole alone Primary outcome: mortality (global mortality, 30-day mortality, 90-day mortality)	↓ LOS in oral step-down group (10 days different, *p* = 0.005) ↓ complications ↓ in-hospital mortality ↓ 30-day mortality No difference in 90-day mortality and relapses Good IDU representation: 15.3% in control group vs. 12.9% in oral step-down group	Promising utilization of oral therapy with mortality benefit and decreased length of stay
N Engl J Med. 2019, 380(5), 415–424. doi:10.1056/NEJMoa1808312. [94] N Engl J Med. 2022, 386(6), 601–602. doi:10.1056/NEJMc2114046. [95]	Randomized-controlled trial Multicenter, unblinded, non-inferiority All patients completed at least 10 days of IV antibiotics Control: continued on IV antibiotics Experimental: transitioned to oral therapy Primary outcome: treatment failure (composite of all-cause mortality, unplanned cardiac surgery, embolic events, and relapse of bacteremia with primary pathogen)	6-month analysis: Streptococci most common pathogen Primary outcome: 12.1% in IV group vs. 9% in oral group (non-inferior) Similar all-cause mortality and safety outcomes Five-year follow-up analysis: Primary outcome: 45.2% in IV group vs. 32.8% in oral group, mainly driven by all-cause mortality	Largest randomized controlled trial for oral stepdown therapy in endocarditis with high follow-up rate Lack of MRSA IE, small number of IDU, and dosing limitations for both IV and PO regimens
Clin Infect Dis. 2023, ciad119. doi:10.1093/cid/ciad119. [96]	Multicenter, retrospective cohort Control: IV-only Experimental: transitioned to oral therapy when meet pre-defined criteria (vs. specific number of days of IV treatment) Primary outcomes: clinical success (defined as being alive, without recurrent bacteremia, and without treatment-emergent infectious complications within 90 days)	Primary outcome at 90 days: 84.4% in IV group vs. 87% in oral group (*p* = 0.66) ADEs: 27.5% in IV group vs. 8.7% in oral group (*p* = 0.004) Good IDU representation: 18% in IV group vs. 37% in oral group Good MRSA representation: 20.4% IV group vs. 34.8% oral therapy group (*p* = 0.004)	Real-life utilization of published literature and national guidance to establish institutional expectations
*Dual therapy for non-HAEK gram-negative IE*			
Microbiol Infect Dis. 2021, 101(3), 115504. doi:10.1016/j.diagmicrobio.2021.115504. [101]	Single-center, retrospective cohort CT: receipt of at least 5 days of two or more antimicrobial agents active against the isolated pathogen MT: received less than 5 days of CT Primary outcome: composite of 60-day all-cause mortality, readmission, or recurrence of bacteremia	Primary outcome: 62% MT vs. 50% CT (*p* = 0.36) No difference in inpatient mortality ↑ median LOS in CT: 20.5 days vs. 12 days (*p* = 0.003)	This study offered evidence for use of MT in non-HACEK gram-negative IE

Abbreviations—CT: combination therapy; IDU: intravenous drug use; IE: infective endocarditis; IV: intravenous; LOS: length of stay; MRSA: methicillin-resistant *S. aureus*; MT: monotherapy; PO: oral.

**Table 5:** Clinical Evidence Summary for Long Acting Lipoglycopeptides.

Citation	Study Design and Methods	Notable Outcomes	Conclusion
Clin Infect Dis. 2018, 67(5), 795–98. doi:10.1093/cid/ciy279. [107]	Retrospective cohort evaluating patients with gram-positive bacteremia and infective endocarditis that received ≥1 dose of DAL	N = 27 Median age = 60 years Microbiological and clinical success = 25/27, 92.6% Average duration of DAL treatment = 6 weeks (range 1–30) Minimal adverse events; 1 patient experienced nausea/vomiting and 1 patient experienced a 2.5× increase in creatinine, which resolved 2 weeks later Type of IE -Native valve = 16 >Surgery = 11 (68.8%) -Prosthetic valve = 6 >Surgery = 1 (16.7%) -Cardiac device related = 5 >Surgery = 4 (80%) Causative Organisms -*Staphylococcus aureus* = 9 -Streptococci = 8 -*Enterococcus faecalis* = 4 -Coagulase negative staphylococci = 7 -*Aerococcus urinae* = 1	High rates of clinical and microbiological success in patients with native, prosthetic, and cardiac device-related infective endocarditis treated with ≥1 dose of DAL
Ann Clin Microbiol Antimicrob. 2019, 18, 30. doi:10.1186/s12941-019-0329-6. [108]	Multicenter, observational, retrospective (14 Spanish hospitals) evaluating the effectiveness of DAL as consolidation therapy for GPC IE and the pharmacoeconomic impact *Note: this study also evaluated patients without IE; all data in this table are specific to patients with IE*	N = 34 Median age = 73 years Clinical cure = 100% -Cure at 12 month follow-up = 96.7% (excludes 1 patient who was lost to follow-up) DAL coverage, median = 14 days (IQR 14–21) Type of IE -Native valve = 32.4% -Prosthetic valve = 44.1% >Surgery = 66.7% -Pacemaker leads = 23.5% >Surgery = 87.5% Valve affected -Aortic valve = 50% -Mitral valve = 23.5% -Tricuspid valve = 2.5% Causative organisms -Coagulase negative staphylococci = 44.1% -MSSA = 20.6% -MRSA = 8.8% -Streptococci = 11.8% -*Enterococcus faecalis* = 8.8% Pharmacoeconomic impact -Reduction in hospital stay, median = 14 days (IQR 7–17); total decrease of 557 days -Cost savings based on 557 hospital days saved = 283,187.45 € ($311,888.50)	DAL is effective for consolidation therapy in clinically stabilized patients with IE. Additionally, it was a cost-effective treatment option reducing hospital stay

**Table 6:** Infective Endocarditis Controversies and Conclusions.

Clinical Questions	Author Conclusions
What beta-lactam antimicrobial should be used to treat MSSA endocarditis?	Either cefazolin or an ASP should be considered first line therapy in the management of MSSA endocarditis; however, cefazolin may be preferred given clinical outcomes and tolerability data.
When should rifampin be added to other active antimicrobials in the treatment of Staphylococcal endocarditis?	Rifampin can be added to another active staphylococcal drug for prosthetic valve endocarditis when there are no contraindicated drug interactions and optimally after blood cultures are negative.
Should aminoglycosides be added to other active antimicrobial(s) in the treatment of Gram-positive endocarditis?	Given increased nephrotoxicity risk associated with use, aminoglycosides should not be considered as first-line therapy in the treatment of IE particularly in those with chronic kidney disease, baseline hearing impairment, and the elderly.
Are daptomycin and ceftaroline used in combination the standard of care for Gram positive endocarditis?	This combination is not yet well established as the standard treatment for all patients. It should be considered as a second line option in the setting of treatment failure for staphylococcal or enterococcal IE, with earlier use potentially associated with increased benefit.
Who can receive oral antimicrobials as stepdown therapy in endocarditis?	Oral antimicrobials can be given as step-down therapy in uncomplicated streptococcal and enterococcal IE. Future research in MRSA IE and IDU will have a significant impact on transitions of care.
Can long-acting lipoglycopeptides be used to treat Gram positive endocarditis?	Long acting lipoglycopeptides can be used for Gram positive endocarditis after completion of at least two weeks of IV antibiotics. It is reasonable to administer a single weekly dose through the end of treatment.

Abbreviations—ASP: antistaphylococcal penicillin; IDU: Intravenous drug users; IE: Infective endocarditis IV: intravenous; MSSA:methicillin susceptible Staphylococcus aureus.

## Data Availability

Not applicable.
