# Correction: Adema et al. Heartbreaking Decisions: The Dogma and Uncertainties of Antimicrobial Therapy in Infective Endocarditis. *Pathogens* 2023, *12*, 703

**DOI:** 10.3390/pathogens13030190

**Published:** 2024-02-21

**Authors:** Jennifer L. Adema, Aileen Ahiskali, Madiha Fida, Krutika Mediwala Hornback, Ryan W. Stevens, Christina G. Rivera

**Affiliations:** 1 Department of Pharmacy, East Carolina University Health, Greenville, NC 27834, USA; 2 Department of Pharmacy, Hennepin Healthcare, Minneapolis, MN 55415, USA; 3 Division of Public Health, Infectious Diseases and Occupational Medicine, Mayo Clinic, Rochester, MN 55902, USA; 4 Department of Pharmacy, Medical University of South Carolina (MUSC) Health, Charleston, SC 29425, USA; 5 Department of Pharmacy, Mayo Clinic, Rochester, MN 55902, USA

**Text Correction**

There was an error in the original publication [1]. A typo in the twelfth sentence of the first paragraph of Section 2.3, “Cefazolin vs. ASPs”, was noted by the authors. It should read “*p* = 0.034” instead of “*p* = 0.34”.

The authors state that the scientific conclusions are unaffected. This correction was approved by the Academic Editor. The original publication has also been updated.
